# A Strategy towards Light-Absorbing Coatings Based on Optically Black Nanoporous Alumina with Tailored Disorder

**DOI:** 10.3390/ma14195827

**Published:** 2021-10-05

**Authors:** Mikhail Pashchanka, Gennady Cherkashinin

**Affiliations:** 1 Department of Chemistry, Eduard-Zintl-Institute, Technical University of Darmstadt, Alarich-Weiss-Straße 12, 64287 Darmstadt, Germany; 2 Institute of Materials Science, Technical University of Darmstadt, Alarich-Weiss Straße 2, 64287 Darmstadt, Germany; gennady.cherkashinin@tu-darmstadt.de

**Keywords:** order–disorder phenomena, anodizing, X-ray photoelectron spectroscopy (XPS), nanostructured materials, photonic light entrapping

## Abstract

This work provides a conceptually new way of thinking about the light-absorbing mechanism in additive-free black porous anodic alumina (black PAA, or b-PAA) layers obtained via “burning” anodizing regime. The new insight into the controllable photonic effects in PAA allows the implementation of the optical blackening method based on the deliberate randomization of the initially well-ordered nanopore arrangement. The proposed black coloration mechanism rests solely on the destructive interference of light after its multiple scattering. Similar effects have been earlier considered for some natural or artificially created biomimetic structures (e.g., the so-called “moth eye effect”, or the coloration mechanism in the Neurothemis tullia dragonfly wings). Comprehensive analysis confirmed that the chemical composition of b-PAA has only a minor influence on the color changes and the optical density increase, and that the light-absorbing properties most likely result from the structural effects. The new functional 2D materials exhibit strong adhesion to aluminum surface, are cost-effective and suitable for application under harsh thermal or UV-light conditions. They are potentially useful for manufacturing of optical devices or heat-resistant coatings in aerospace technologies, as well as solid supports for biological filtration and fluorescence imaging.

## 1. Introduction

Optical and photonic properties of porous anodic alumina (PAA) layers have gained considerable attention of late [1]. Although pure alumina is known to be a reflective, transparent and intrinsically colorless insulating material with a large band gap, the absorption of light of certain wavelengths can be achieved in PAA due to photonic effects and destructive interference [2]. Materials which normally show a high reflectance across the whole range of visible wavelengths may often also exhibit structural coloration or even deep blackening due to light entrapping upon corresponding texturing of their surface. For example, polished mirror-like cutting edges of thin metallic blades (for surgical scalpels, razors etc.) can absorb light and form a featureless dark surface when aligned and stacked together. This so-called “black velvet” effect is used in industry for the sharpening quality control and detection of defects which appear on the surface as white spots. In the case with intrinsically transparent PAA, the minor absorption of light (which excites atom vibrations) will be additionally combined with the diffusion of multiply reflected light waves within the layer, where particular wavelengths can be also cancelled out due to the destructive interference effect. Random distribution of scattering elements (i.e., cylindrical nanopores in PAA) may lead to the strong change of optical properties of a photonic system [3]. While the long-range ordered photonic crystals exhibit a low density of optical modes within a narrow frequency window (formation of the well-defined photonic band gap in naturally occurring PAA [4,5]), the random systems could hypothetically show a strong multiple scattering and absorb in a broader wavelengths interval (light entrapping). This difference between the optical properties of photonic crystals and disordered photonic materials follows from the photonic band gap formation mechanism: for certain wavelengths of radiation, refractive indices, and spacing between the light scattering elements, complete cancellation of the waves (destructive interference) reflected from different interfaces can occur in all directions, so that particular wavelengths are not transmitted by the layer [6]. Thus, variable spacing may lead to destructive interference in a broader range of visible wavelengths, and enhance the light absorption. It has been previously observed that the randomness in morphology leads to strong multiple scattering, which forms an efficient trapping mechanism to localize light and obtain strong absorption [7]. Theoretically, random 2D photonic PAA with different introduced degrees of disorder should demonstrate the absorption in a larger wavelengths interval. Developing this concept can ultimately enable even manufacturing of “black porous alumina” (b-PAA) photonic films adhered to a metallic substrate. This proposed black coloration mechanism is identical to the one that already exists in natural super-black surfaces, or in some artificially created biomimetic structures. For instance, a similar approach (the electrochemical etching of nanopores) is widely used for fabrication of so-called porous silicon (p-Si) or black silicon (b-Si) [8,9]. The surfaces of these materials demonstrate either vivid colors (p-Si), or a blackened surface (b-Si) with extraordinarily low reflectance over a broad spectral range due to the strong photonic light-trapping. Functional properties of b-Si arise due to the bio-inspired antireflective surface morphology (nanoscale pillar array) based on the so-called “moth eye effect”. The self-ordered PAA films are the most obvious candidates for manufacturing inverse (i.e., consisting of holes in dielectric medium) light-entrapping surfaces. However, to the best of our knowledge, there are currently no reports on such a line of investigation in the literature, and the knowledge in this field still remains rather fragmentary.

Black coloring of PAA films is frequently observed when the phenomenon called “burning” (that is a strong increase of the electrical current, accompanied by local etching and distortion of the ordered structure) occurs during the electrochemical synthesis [10]. However, the nature of this coloring remains relatively unexplored. It has still not been clarified whether it results from a chemical impurity, the increased optical density, or photonic effects. Kikuchi et al. have associated the appearance of b-PAA during the anodic oxidation of aluminum in cyclic oxocarbon acids with the local thickening of the growing oxide film [11]. However, thick oxide spots caused by burning in phosphoric or phosphonic acid electrolytes resulted in the appearance of whitish color [12]. Thus, the formation of b-PAA cannot be ascribed solely to the increased thickness of the oxide layer and therefore also the increased optical density. Nonetheless, these different observations can be readily interpreted from the photonics standpoint: for the light diffraction and photonic effects to occur, the desired periodicity in the refractive indices variation (which is equivalent to the interpore distance *D*_int_ in PAA) must be half the wavelength of the electromagnetic wave [1]. The PAA cell size obtained from oxocarbon acids was 200–450 nm, which means that the doubled values (400–900 nm) covered the whole visible region (400–700 nm) [11]. At the same time, the interpore spacing obtained from phosphonic acid electrolyte was 370–440 nm, and the doubled values yield 740–880 nm, which is already beyond the visible region for a human eye [12]. It must also be mentioned that the light waves which participate in the destructive interference do not necessarily need to be reflected from directly neighboring pores. Thus, the phenomenon of photonic light absorption can very likely take place in the cases with *D*_int_ significantly smaller than 200 nm as well.

Herein, we report fabrication of light absorbing inverse photonic systems that have not been previously systematically studied–namely, randomly patterned b-PAA. We demonstrate that uniform large-area black coatings can be achieved using pigment-free deliberately randomized nanoporous alumina. These films exhibit strong adhesion to the Al surface, are mechanically robust, cost-effective and suitable for application under harsh conditions such as elevated temperatures or irradiation by UV-light. Therefore, they have numerous advantages over traditional carbon-based materials and can be used as alternatives to replace the light-absorbing carbon nanotube (CNT) arrays. In addition, the refractive index of PAA can be adjusted close to 1—that of air (the values reported so far are ≤1.25) [13,14]. This makes tailored PAA structures very promising candidates for light absorption because incident light will not “see” the barrier of the surface, and will be more preferably absorbed than reflected. Blackened alumina is potentially useful for many areas including construction of optical devices and aerospace technologies. For instance, b-PAA can find application as a resistant darkening or heat-eliminating coating which is compatible with lightweight and durable Al-based alloys. In our present study, numerous analytical methods confirm that the light absorbing properties of b-PAA very likely arise from the photonic effects, but not from the variable non-stoichiometric composition or occasional admixture, such as carbon, sulfur, copper-related phases, or fine-dispersed inclusions of metallic aluminum which could possibly be impregnated into the growing alumina under high current densities.

## 2. Materials and Methods

As the starting experimental conditions for the present investigation, anodizing regimes from previous work dealing with diluted sulfuric acid electrolytes were employed [15]. In contrast to the earlier study where voltages up to 28–29 V were applied and yielded highly transparent or translucent PAA samples, we presently apply higher 30–34 V potential differences and observe further gradual evolutional changes towards novel homogeneous opaque black b-PAA coatings. The anodic oxidation of aluminum sheets (PURALUX^®^, purity 99.93%, thickness 1 mm) was performed in 0.1 M and 0.2 M aqueous H_2_SO_4_ solutions under potentiostatic conditions using a Toellner TOE 8952 power supply (maximum output 40 V and 3 A per channel). High-purity sulfuric acid (95–97%) was purchased either from Grüssing or from VWR. Solutions were prepared using deionized water (*σ* ≈ 2 μS/cm). A thermostatic system (Julabo F-12MA) kept the temperatures of the open baths at 0.5 ± 0.3 °C. The bath temperatures could temporarily change to 2.9–4.5 °C in the case of a violent “aluminum burning” process at rapidly increased current densities, but gradually went back to normal upon the increased PAA layer thickness and a larger ohmic resistance. The electrolytic baths of a comparatively large 9 L volume were also vigorously agitated with a magnetic stir bar for the effective heat elimination from the electrodes. The total surface area of an anode was approximately 30–35 cm^2^ (the front-, the backside, and the lateral faces summed up together). After the first anodizing for 6 h, the working electrodes were immersed into the etching solution (24 g of K_2_Cr_2_O_7_ and 59 mL of 85 wt% H_3_PO_4_, filled up to 500 mL with distilled water and kept at ≈60 °C on a hot plate). After such preparation of the substrate texture, the main oxidation was performed for 20 h. Resulting black alumina layers (b-PAA) were separated from metallic substrates using the polarity reversal method, which is described elsewhere (the method was proven to leave no metallic residues on the bottom side of the alumina layers) [16,17]. Free-standing b-PAA films were thoroughly rinsed with deionized water and stored in a drying oven at 60 °C.

The samples were characterized by HRSEM using a Philips XL-30 FEG electron-scan microscope coupled with an energy-dispersive X-ray (EDX) analyzer operated at 20–25 kV; samples were mounted on conductive carbon-rich polymer films and sputtered with a 4.5 nm layer of Pt/Pd alloy. The selection of long-range ordered and disordered areas in HRSEM images was performed manually using standard image-editing software. Every individual pore coordinated with 6 nearest neighbors was marked in cyan, whereas five-, seven-, or eight-fold coordinated pores and other defects in the hexagonal arrangement were marked in red. As the number of color-coded pores increases, they merge into the corresponding well-ordered and disordered domains that cover the entire area of an HRSEM micrograph. Infrared spectra were taken by the KBr-pellet method on a Thermo Scientific Nicolet 6700. Micro-Raman spectroscopy was performed on a Horiba Jobin-Yvon HR800 spectrometer using 514 nm laser wavelength. X-ray diffractometry analysis (XRD) of the species was carried out on a Stoe and Cie StadiP diffractometer in Debye–Scherrer geometry using Cu K_α1_ radiation (*λ* = 1.541 Å) with a Ge (111) monochromator. TGA data were obtained on a Netsch STA 449C Jupiter at a heating rate of 10°C min^−1^ from ambient temperature to 1300 °C, using synthetic air atmosphere (flow rate 75 mL min^−1^). X-ray photoelectron spectroscopy (XPS) was applied to study chemical composition of the coating. The photoelectron spectra were collected by using a PHI 5000 VersaProbe spectrometer (Physical Electronics) and a monochromatic Al Kα (*hν* = 1486.7 eV) source at an electron escape angle of *θ* = 45°. The charging effect occurring in insulators was compensated by using a low-energy electron gun. The removal of a thin near-surface layer for the in-depth XPS analysis was performed by means of sputtering with Ar^+^ ions. From the known duration of Ar^+^-ion sputtering and typical etching rates, the estimated average thickness of the removed surface layer was approximately equal to 30 nm. All samples were mounted between a metallic sample holder and a perforated tantalum foil, which had small windows bounding the analyzed sample areas. During Ar^+^-sputtering, some of the incident argon ions collided with the surrounding foil and small amounts of Ta could be dispersed onto the surface of the examined PAA and b-PAA samples. This accidental contamination, however, by no means disturbs the sample analysis because Ta-signals do not interfere with any signals of interest. The reflectance of PAA and b-PAA samples was recorded on a UV/Vis/NIR spectrometer Lambda 900 from Perkin Elmer.

## 3. Results and Discussion

**Figure 1:**
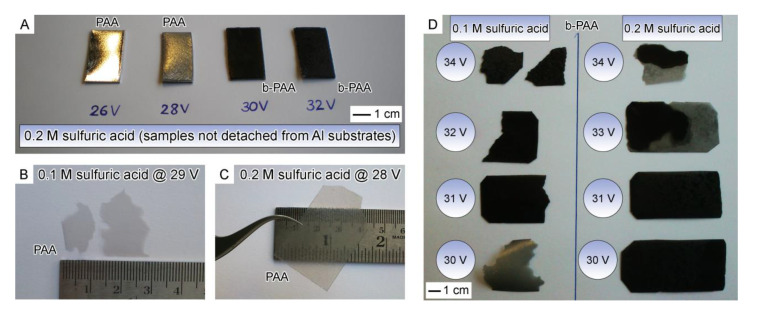
Gradual color and transparency changes in anodic alumina upon the stepwise increase of anodizing voltages is illustrated by samples obtained from 0.2 M H_2_SO_4_ electrolyte (**A**) together with the underlying polished metallic substrates. The visual appearance of highly reflective Al-surface beneath alumina layers in (**A**) clearly shows how their optical properties change from highly transparent at 26 V to translucent and moderately lusterless at 28 V, and further to deep black and opaque at 30 and 32 V. All transparent PAA (**B**,**C**) and opaque black b-PAA films (**D**) obtained from 0.1 M and 0.2 M H_2_SO_4_ electrolytes at varied voltages of 29–34 V and 28–34 V, respectively, can be detached from metallic substrates and obtained as unsupported (free-standing) samples by the polarity reversal method.

The uniform visual changes in the anodic alumina layers achieved upon progressively increased anodizing voltage are exemplified in Figure 1A by the PAA and b-PAA samples formed in 0.2 M electrolyte at 26–32 V with 2 V increment. The highly reflective metal surface can be easily seen through the deposited boehmite (*γ*-AlOOH) layer at 26 V. Before the transition to opaque black films at higher voltages occurs, the system passes through the diffuse reflection state which is observed at 28 V. However, at the current stage of such visual comparison by the naked eye, the diffuse reflection can be associated either with the gradually increasing disordering in the nanopore layout, or the irregularity in the constituting microcrystallites orientation within pore walls, or the increased surface roughness, or a cumulative effect of all these factors.

Figure 1D shows b-PAA films obtained in two series of experiments using 0.1 M and 0.2 M sulfuric acid electrolytes after their detachment from Al substrates using the polarity reversal technique. As Figure 1D indicates, both types of optically black alumina layers demonstrate conformity with each other in deep coloration. These results speak in favor of the applicability of the method for intermediate acid concentrations as well, or possibly even for their slightly expanded range. However, the films from 0.1 M electrolyte exhibit a smoother transition from translucent films to opaque black than their counterparts from 0.2 M acid—namely, the sample obtained from 0.1 M H_2_SO_4_ at 30 V shows intermediate visual properties with residual translucence and the beginning of darkening, which can preliminarily be explained either by a slower film growth rate during this experiment, or by only moderate change of compositional or morphological properties (residual regularity in pore layout, corresponding to the translucent state). Moreover, the specimens obtained from 0.2 M electrolyte at 33 and 34 V show more prominent signs of the local electric current concentration and uneven thickness across deposited layers. This emerging inhomogeneity in film electrodeposition (and therefore also minor optical inhomogeneity) manifests the transition to the uncontrollable “aluminum burning” regime. Such regime means locally enhanced dissolution of the metallic substrate (which can be accompanied by accelerated oxide growth, but not necessarily) and possible approach to the critical voltage, after which the electrical breakdown may occur [10]. Under such conditions, testing voltages above 33–34 V does not appear practical for the purpose of our comparative study, although the b-PAA samples obtained from 0.1 M solution at 34 V are still rather uniform. It needs to be mentioned here, however, that the achieved homogeneous and reproducible blackening of anodic alumina films over large surface areas of tens of cm^2^ is a significant practical advance, which is now feasible due to the new found mild anodizing conditions using highly diluted H_2_SO_4_ electrolytes. The appearance of dark local spots on PAA has been previously also known as a manifestation of spontaneously occurring “aluminum burning” which often disturbs the self-ordering processes typically studied in PAA systems. The “burning” regime can boldly be called the most chaotic and uncontrollable, and the least explored to date. It would be no exaggeration to call acquiring control over such complex phenomenon, and making practical use of it, a significant development in anodizing techniques. Since stabilization of any unstable states and controlling of uncontrollable regimes is generally considered to be the primary task of the modern research in the area of self-organization in electrochemical systems [18], our discovered new anodizing regimes and evenly distributed “moderate burning” on aluminum surface can be presumably useful not only for applied, but also for fundamental research purposes.

In order to provide a prompt visual test for the earlier proposed (by Kikuchi et al.) idea that the black coloring may appear owing to the local thickening of the growing oxide film under high current density conditions [11], several translucent PAA layers (individual thickness of ≈70 µm) were superimposed and compared with a black sample. This was performed to achieve the same total thickness of the stacked material as that of the single b-PAA film. As can be observed from the Figure S1 presented in the Supplementary Materials, the only cumulative effect of such model system is the drastic loss in transparency (which is obvious due to the increased optical path within the scattering media), but no visible changes towards optically black state took place. Thus, the film thickening is very likely not the major factor responsible for the deep black coloration. It has to be accompanied by other transformations, such as compositional or morphological (e.g., alterations in the in-plane PAA structure).

**Figure 2:**
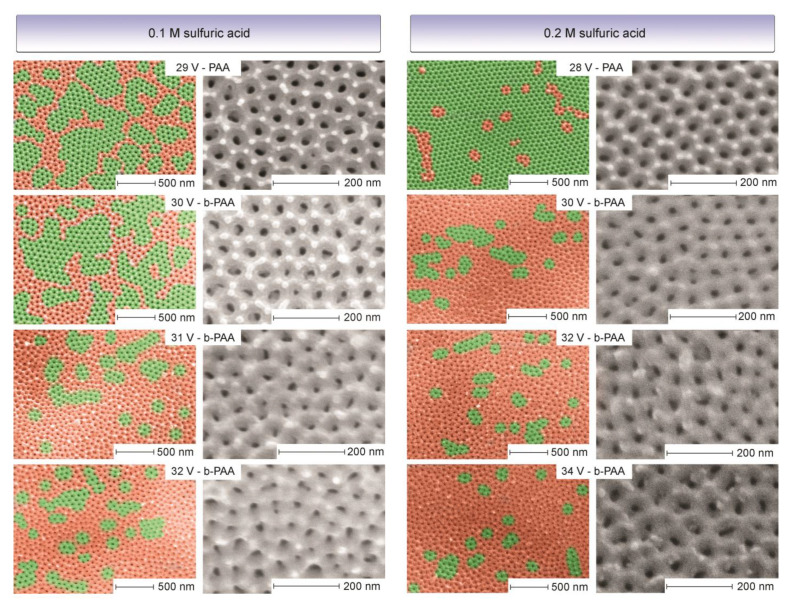
The reverse evolution of the long-range ordering at the nanoscale level in anodic alumina films upon the increase of anodizing voltages and transition from transparent porous anodic alumina (PAA) to optically black (b-PAA) layers in 0.1 M and 0.2 M H_2_SO_4_ electrolytes (all samples are analyzed underneath a scanning electron microscope (SEM) at consistent 80,000× and 350,000× magnifications). The hexagonally ordered photonic crystal-like areas are color-coded as green; the disordered areas containing randomly coordinated pores are color-coded as red. The apparent difference can be observed between macroscopically transparent PAA (0.1 M @ 29 V and 0.2 M @ 28 V) where the long-range ordered domains are prevailing, and the b-PAA samples that are predominantly disordered. The mixed semi-ordered character of the sample corresponding to 0.1 M @ 30 V may serve as an explanation of the transitional (semi-translucent and semi-blackened) macroscopic visual appearance in Figure 1.

One striking difference is apparent between the transparent (or translucent) PAA layers and the b-PAA samples when their scanning electron microscopy (SEM) images are compared (see Figure 2). While the PAA samples are predominantly highly ordered and consist of large flawless domains containing six-fold coordinated pores without significant *D*_int_ variations, their b-PAA counterparts are almost completely random with only small-size coherent areas included. This character of development is very pronounced in both cases, irrespective of the electrolyte concentration employed. In the light of this evidence, it is obvious that the idea about the photonic nature of black coloration in anodic alumina films is rather viable (until it has been shown otherwise). Furthermore, the semi-ordered structure within this sequence with still large embedded coherent domains, i.e., the semi-opaque layer formed in 0.1 M electrolyte at 30 V, supports the idea that gradual randomness introduction can provide control over the optical darkening tuning. However, since the correlation between the morphological randomness and blackening does not automatically mean their causation, the following hypotheses have to be checked to exclude any chances that the light absorption results from the chemical composition features:

- Amorphous carbon admixture, which is highly unlikely from the experimental conditions. The decomposition of amorphous carbon in air atmosphere occurs at 315–355 °C [19], which should also lead to visible color changes. In addition, Raman spectroscopy is a powerful tool for detection.
- The presence of black cupric oxide (CuO) which may be associated with the copper-containing alloy from the electrode clamps. This explanation is also very unlikely because the black color appearance under high-current–density regimes usually has good lab-to-lab reproducibility irrespective of the electrode clamp materials used. Besides, the electrodeposition of Cu oxides and hydroxides typically requires alkaline electrolytes [20].
- Inclusion of metallic aluminum (Al^0^) fragments, which could be entrained by fluid flows together with colloidal alumina particles at high currents and incorporated into the growing pore walls (see the nanoconvective model of PAA formation) [21]. This explanation would be the most plausible one because aluminum is always present in any electrochemical PAA system as the substrate material regardless of the electrolyte used.

If none of these proposed possibilities are confirmed with the numerous analytical methods used in this work, this will indirectly (by the exclusion method) support the idea of the photonic nature of b-PAA.

**Figure 3:**
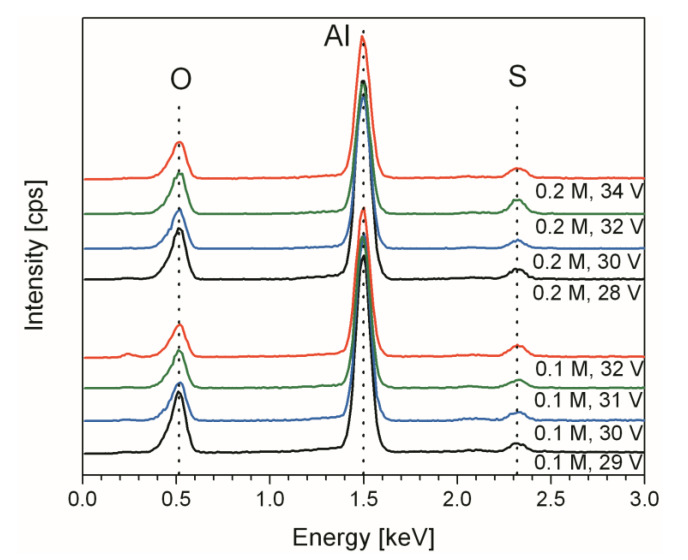
Comparison of energy-dispersive X-ray (EDX) elemental analysis of free-standing translucent PAA (obtained from 0.1 M H_2_SO_4_ @ 29 V and from 0.2 M H_2_SO_4_ @ 28 V) and opaque black (obtained at 30–34 V from both used electrolytes) b-PAA layers.

Figure 3 shows the comparison of the EDX analysis for the PAA layers obtained at different voltages, including the conventional colorless samples and the new b-PAA samples. It is evident from the EDX-spectra that all the specimens have qualitatively identical composition irrespective of their visual appearance. No elements other than aluminum, sulfur and oxygen can be detected in all the examined cases. Whereas efficient detection of small amounts of carbon might be difficult because of the limited sensitivity of the method to light elements (the lightest one that can be detected by EDX spectroscopy is boron, *A*_r_ = 10.81), the conducted analysis is still very helpful for the detection of impurity phases related to other metals besides aluminum (e.g., Cu in black CuO). It is important to note, however, that spectroscopic tools are typically limited in their ability to characterize porous materials only in the vicinity of the surface (the EDX analysis depth depends on many parameters, such as the acceleration voltage, free path of the incident electrons in the dense sample etc., but in our case we can safely assume the maximum analysis depth to be about 2–3 µm). The composition monitoring was performed by collecting EDX data from multiple spots on PAA and b-PAA samples, and the standardless quantification method demonstrated moderate Al-content variation in the range between 33 and 38 at % in all specimens, irrespective of the concentration and voltage used for the synthesis. Taking into account the stable regime of long-continued pore growth and the persistent electrodeposition conditions maintained during the entire procedure, a largely invariable composition throughout PAA along the pore growth direction can be expected. This finding (no significant fluctuations of the Al concentration among different analyzed samples, or also no large increase with higher applied voltages) allows excluding the formation of metallic aluminum (i.e., Al^0^) inclusions as a possible factor of the anodic alumina darkening. The support for this conclusion can also be found in the earlier report of Durtschi et al., where EDX-studies demonstrated moderate variations of the constituting elements concentrations (e.g., 33.0–37.8 at % for aluminum) in the cross-section of a single PAA sample [22]. It needs to be mentioned that Durtschi et al. analyzed the composition of the commercial Anopore^TM^ membranes (Whatman Inc.( Little Chalfont, UK)), which are initially colorless (whitish), and those authors needed an electroless deposition of nickel-boron nanoparticles into the pores for increasing filter optical absorption and obtaining a black PAA membrane. The two-step method from that work was designed for improving the properties and applicability of PAA as a solid support for biological filtration and solid phase imaging. In our current study, we are obtaining identical optically black PAA filters in just one single step, and no further membrane modification process for fluorescence imaging would be required. There is, however, one interesting systematic change in the b-PAA composition with the increased applied voltage during the synthesis which is revealed by the EDX analysis–namely, the sulfur content increases from approximately 2.2 at % (or 3.4 wt %) in translucent membranes to about 3.7 at % (or 5.9 wt %) in optically black samples (the measuring error of the standardless quantification method is around 1 wt %). At this stage of the investigation, it can be supposed that the b-PAA darkening mechanism is probably associated with the sulfur-containing impurity (by analogy with silicate glasses which can absorb in the UV wavelength region of the spectrum due to the presence of impurity, whereas pure silica is transparent for the UV-light).

**Figure 4:**
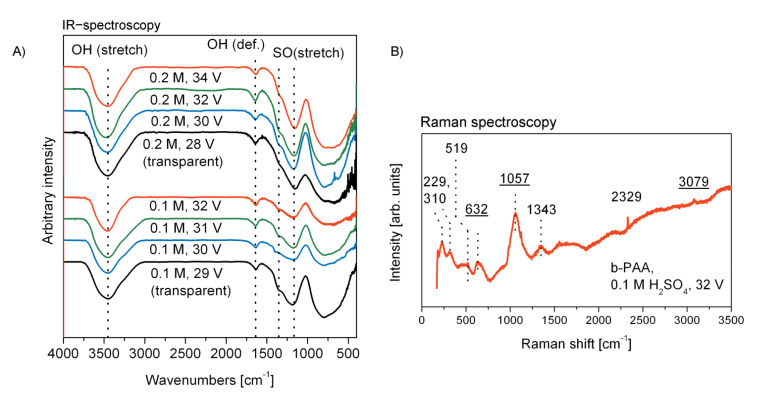
Summary on the vibrational spectroscopy data obtained for PAA and b-PAA synthesized in this work (all specimens are detached from substrates). Left, (**A**): comparison of the infrared (IR) spectra for translucent PAA (obtained at 28 and 29 V) and opaque b-PAA (30–34 V) layers from both studied electrolytes. Right, (**B**): the typical Raman spectrum features of a representative unsupported b-PAA sample (obtained from 0.1 M H_2_SO_4_ at 32 V). The underlined wavenumbers correspond to the visible Raman peaks that have been previously also reported for translucent PAA. Both the IR and Raman spectroscopy methods confirm absolute absence of carbon- or copper-related admixture which could cause the black coloration.

In Figure 4 (on the left, A), the infrared spectroscopy (IR) characterization of the PAA and b-PAA samples is presented for comparison. Typically for slightly acidified alumina, all samples demonstrate hydroxylated and hydrated surface, which manifests itself through the absorption in the regions corresponding to OH stretching (usually between 3650 and 3200 cm^−1^) and deformation (at 1640–1615 cm^−1^ for crystal water in solids) vibrations [23]. The characteristic series of bands in the region 1420–1020 cm^−1^ commonly corresponds to the stretching vibrations of S=O double bonds [23]. The intensive two signals with maxima at 1350 and 1170 cm^−1^ can be assigned to the asymmetrical vibrations in −SO_2_− groups, which are usually observed in the regions 1450–1300 and 1210–1150 cm^−1^. [23] All samples also show the characteristic signals for the S–O bonds stretching vibration, which are known to be located at 740–720 and 710–690 cm^−1^ [23]. Together with the broad bands of the out-of-plane vibrations of OH-groups at 750–650 cm^−1^, these S–O bond signals contribute into the wide absorption at 1015–400 cm^−1^. The obtained IR-spectroscopy data supports the information obtained in EDX-analysis (Figure 3) and confirms that the optically black layers are free from any graphitic impurity, which would generate a structureless signal between 1650–1580 cm^–1^ corresponding to the stretching mode of the conjugated C=C framework of graphite.

In order to supplement the IR-spectroscopy analysis and to complete the comprehensive vibrational spectroscopy characterization of the samples, a typical Raman (combinational scattering) spectrum of deep-black b-PAA layers after their detachment from the aluminum substrate is demonstrated in Figure 4 on the right (B). The characteristic features of b-PAA Raman spectra are examined here in the case of the sample obtained from 0.1 M H_2_SO_4_ electrolyte at 32 V. The low-intensity signals located at 519, 632, and 3079 cm^−1^, as well as the prominent signal centered at 1057 cm^−1^ have been previously reported for standard translucent PAA [24]. The signal at 632 cm^−1^ can be specifically attributed to the ν_4_ mode of the SO_4_^2−^ anion from electrolyte admixture [25]. The band at 311 cm^−1^ belongs to the *ν*_g_ vibration mode of water [25]. The signal at 519 cm^−1^ has been also interpreted as belonging either to *ν*_T_ H_2_O or to *ν*_4_ SO_4_^2−^ mode. Raman spectra indicate that the opaque black PAA layers contain no unintentional impurity which could directly cause such drastic color changes in comparison with the conventional translucent films. For instance, completely amorphous carbon admixture would result in one broad signal with the maximal intensity value around 1500 cm^–1^, which usually splits into two signals located at around 1300 and 1600 cm^–1^ upon the partial ordering of the carbon into a graphitic structure (the precise signal positions, as well as their relative intensities depend on the number of graphitic layers) [26]. These two signals correspond to the modes called D (for “disordered”) and G (for “graphitic”), and are typically accompanied by the 2G signal at around 2700 cm^−1^, which is also not observed in our spectrum in Figure 4B. Thus, any carbon admixture causing the black coloring can be excluded. Similar considerations can be applied to exclude the black CuO (cupric oxide) impurity as well. The presence of copper-related phases could be assumed taking into account the copper-containing material of the electrode clamps and corrosion and transport phenomena in aqueous electrolyte bath under high current density conditions. In addition to the observed bands in Figure 4B, cupric oxide would add another well-resolved signal at 350 cm^−1^, which cannot be observed in our samples [27].

As mentioned before, there is uncertainty about whether the increased optical absorption may come from the higher sulfate impurity content in b-PAA in comparison with the conventional transparent films, or the optical changes are predominantly caused by the morphological changes in a PAA photonic lattice. An optimal choice to confirm higher sulfate content in b-PAA would be to supplement the spectroscopy with other methods which are capable of characterization not only below the surface, but also in the bulk of the material. Therefore, we conducted a comparative thermogravimetric study of a representative b-PAA sample from this work (synthesized in 0.1 M H_2_SO_4_ at 31 V) and the highly transparent PAA film synthesized using the method reported in the classical work of Masuda et al. [28]. We have taken one of the most conventional and well-studied transparent anodic alumina specimens as a reference for comparison, which is obtained from a higher concentrated electrolyte (0.3 M H_2_SO_4_) at a slightly lower voltage of 27 V. Thus, the chemical nature of the impurity is in both cases the same, but the quantitative difference in impurity content is possible after modifying the electrodeposition conditions. Since determining of higher sulphate content alone does not confirm any interconnection with the black coloring (i.e., correlation does not mean causation), we also conducted a series of thermal treatment experiments and searched for a relationship between the changes in visual properties and the stages of the b-PAA thermal evolution. By this approach, we can determine more safely whether black coloring is related to the sulfate contamination, or rather to other parameters which also undergo changes within different temperature intervals.

**Figure 5:**
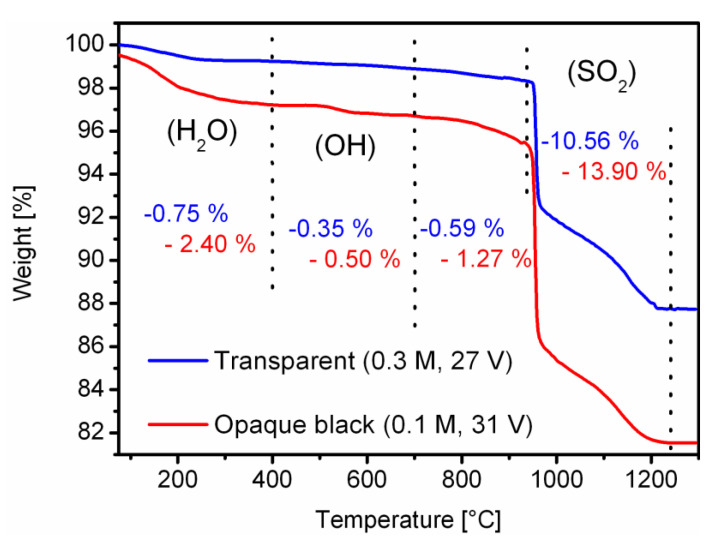
A comparative thermogravimetric analysis (TGA) of transparent PAA and opaque black b-PAA samples obtained from sulfuric acid electrolytes under moderate applied voltages.

Figure 5 displays the results of the comparative TGA analysis of the b-PAA film and the conventional transparent PAA layer obtained by Masuda’s method (samples were detached from aluminum substrate prior to examination; microscopic characterization of the trivial highly ordered and transparent PAA from 0.3 M H_2_SO_4_ is not included into this work; however, a photograph presenting its macroscopic visual appearance is demonstrated in Figure S2 of the Supplementary Materials file). Both TG curves exhibit a similar multi-step decomposition process, but with apparent quantitative differences. The interpretation of the mass-loss events in PAA films can be found in the extensive study reported by Mata-Zamora and Saniger, where the temperature intervals for dehydration, dehydroxylation and decomposition of sulfate anions during the TG measurements were precisely determined [29]. During the first mildly sloping thermogravimetric event which is over at approximately 400 °C we can clearly see the difference in the mass-loss between two compared samples due to the removal of absorbed and coordinated water. This difference can indirectly confirm a more acidic character of the b-PAA sample because additional Lewis acid sites are efficient host centers for Lewis bases and can presumably facilitate water absorption. The next minor decomposition step that continues up to approximately 700 °C has been attributed to the slow dehydroxylation process. In this region, the b-PAA sample also demonstrates observably more acidified surface with a larger number of OH-groups than the conventional transparent reference sample. Interestingly, black alumina shows a noticeable mass loss in the next region between 700 °C and 940 °C which was previously considered in the literature as the plateau of comparative thermal stability [29]. This new finding, however, can be explained by elimination of the remaining hydroxyl groups from the surface. An earlier infrared spectroscopy investigation has demonstrated that approximately 10% of the alumina surface is still covered by the residual OH groups under vacuum conditions at the temperature of 650°C, which means that the coverage under ambient pressure and humidity can be even higher [30]. This quantitative distinction of the new b-PAA sample allows us to update the knowledge about the thermal decomposition of non-stoichiometric anodic alumina and reconsider the temperature intervals in which the dehydroxylation process is completely finished. The final steep mass-loss event which starts at 940 °C and is finished by 1240 °C corresponds to the decomposition of sulfate and is thus the main focus of our TG studies. As can be seen in Figure 5, b-PAA contains about 31.6 wt % more sulfate impurity relative to the transparent reference sample. Thus, the quantitative TGA results are in full accordance with the spectroscopic data and confirm the anionic impurity content increase in the black opaque samples with respect to the conventional transparent and semi-transparent alumina.

**Figure 6:**
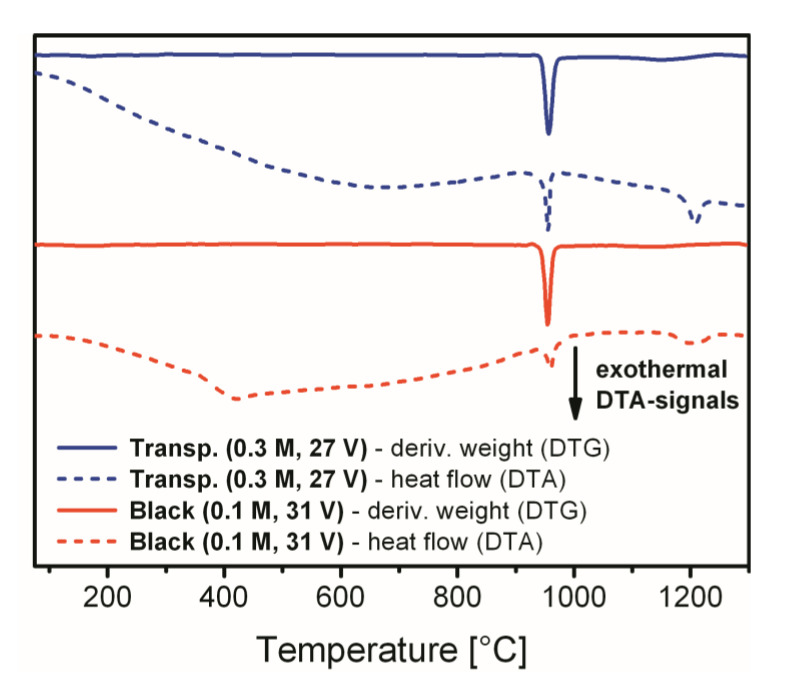
Comparison of DTG and DTA curves obtained for transparent PAA (shown in blue) and opaque black b-PAA samples (shown in red).

Figure 6 presents the DTG and DTA characterization curves for both compared samples, which help to specify characteristic temperatures of the mass loss events more precisely. In addition, DTA allows for identification of the thermal processes which are not necessarily associated with the mass changes in TGA/DTG. The DTG peaks maxima that correspond to the largest mass percentage change during the sulfate decomposition step are located at 955 °C for both analyzed specimens. Other DTG peaks that correspond to minor mass loss events are not prominent in Figure 6. As we can see from the DTA curve corresponding to the transparent alumina sample, the endothermic processes of dehydration and dehydroxylation are practically merged into a single smooth curve of constant slope up to 670 °C. At the same time, the broad endothermal dehydration signal for black alumina has a sharp termination at around 420 °C. This feature visually appears in Figure 6 as an exothermal signal and is followed by a very slight endothermic curvature again up to 680 °C which denotes continuing minor process of the OH-groups elimination from the surface. The exohermal signal at 420 °C very likely corresponds to the transformation of amorphous boehmite into *γ*-Al_2_O_3_. Based on neutron diffraction data, Paglia et al. have previously determined that the onset of the boehmite to *γ*-Al_2_O_3_ phase transformation occurs at 400 °C, and the conversion is complete by 500 °C [31]. Thus, the peak centered at 420 °C very likely corresponds to the lattice change and phase transformation. It needs to be noted, however, that dehydrated boehmite undergoes several transformations into a number of intermediate phases (such as *γ*, *δ*, and *θ*) before the final corundum phase is achieved [32]. Therefore, optical changes in alumina can be also associated with other multiple phase transitions in the interval approximately between 350 and 1000 °C. The intense exothermal DTA signal in Figure 6 centered at 960 °C matches the sulfate decomposition region for both examined samples in Figure 5. The last exothermal peak of low intensity with the maximum at 1200–1210 °C corresponds to the final phase transformation of *γ*-alumina into corundum. This transformation temperature may have slight variations depending on the presence of impurities and on the thermal treatment procedure [29]. It can be also concluded from the DTA data in Figure 6 (to the extent of this analytical method precision) that the black alumina sample is free from any noticeable amount of the metallic Al^0^ that could give rise to the darkening and loss of transparency. The reaction of metallic aluminum with the oxidizing atmosphere is highly exothermic, which would give an additional prominent signal in the DTA curve. The absence of such a signal gives reason to think that metallic inclusions are not present in the bulk of the analyzed b-PAA.

In order to follow the visual changes in b-PAA after different stages of thermal evolution, a representative sample (from 0.1 M electrolyte at 31 V) was calcined at different constant temperatures. The choice of treatment temperatures was determined by the following criteria:

- At 450 °C, the dehydration and the decomposition of any accidental organic admixture and amorphous carbon should be finished (decomposition of carbon in a more crystalline form would require a higher decomposition temperature, but we can rely on Raman spectroscopy and state the absence of any graphitic fragments).
- At 550 °C, the first known phase transformation of boehmite AlO(OH) into *γ*-Al_2_O_3_ is already complete. However, multiphase composition and coexistence of microcrystalline *γ* and *θ* aluminas in various proportions can be expected at higher temperatures up to 1000 °C, and pure single-phase *γ*-alumina exists up to 1200 °C [29], before it undergoes the final transformation into corundum.
- The temperature of 660 °C corresponds to the aluminum melting point, which should affect metallic residues within the sample. In addition, thermal oxidation of metallic aluminum into alumina takes place at 610 °C and has to be complete after annealing the sample at 660 °C for 2 h [33]. According to an earlier investigation, oxidation of metallic aluminum in oxygen atmosphere occurs at a slow rate already at the temperatures as low as 400 °C, and more rapidly at 500–600 °C [34]. However, somewhat counterintuitive results were reported for this kinetic study because the reaction presumably depends on the number of surface defects on aluminum, as well as on the naturally existing protective alumina layer condition (amorphous to crystalline).
- The last annealing was performed at a relatively high temperature of 850 °C, but still significantly below the sulfate-decomposition onset point in TGA.

**Figure 7:**
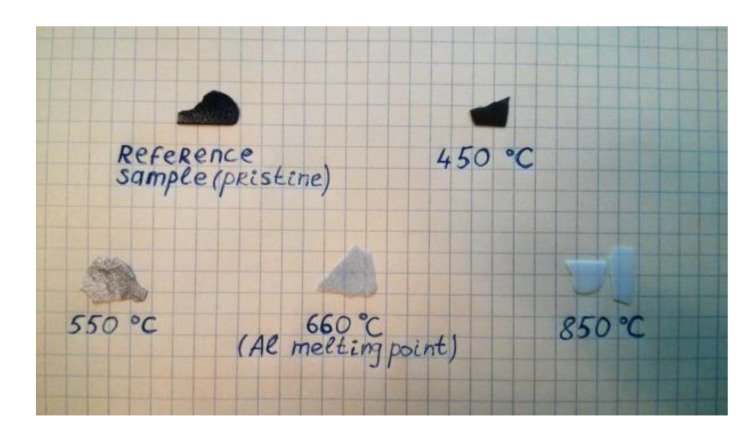
b-PAA samples after thermal treatment at selected elevated temperatures. No correspondence between black color extinction and C-, Al- or S-related impurity oxidation has been confirmed. However, the gradual color transition correlates well with a series of alumina phase transformations extended from 550 to 850 °C.

The results of the thermal treatment experiments are shown in Figure 7. As can be concluded from the examination by the naked eye, there is no correlation between the amorphous carbon degradation temperature and visible color changes. The first disappearance of the deep black color with only remaining dark-grey hue can be observed at 550 °C, which correlates well with the first phase transformations in alumina, but is still below the oxidation temperature of metallic aluminum (if any was contained in the sample). The gray shades also continue to lighten gradually at higher temperatures, but no complete gray color extinction can be observed at 660 °C where metallic aluminum clusters oxidation was expected. The darkening effect fully disappears and the b-PAA layers turn into optically white material at 850 °C, i.e., after a series of phase transitions in anodic alumina. Since the first signs of sulfate decomposition appear at 940 °C, our thermal evolution test suggests that the black coloration cannot be attributed to the anionic impurity content. The most likely reason for color disappearance is the phase transformation in aluminum oxide-hydroxide and corresponding changes in photonic effects–namely, boehmite has an anisotropic refractive index value equal to 1.64–1.67 (the material is, however, amorphous, and variations due to sulfate content are also possible), whereas the converted stoichiometric alumina has a refractive index of 1.76–1.77. Such change of the optical properties also changes the optical path lengths of the propagating light waves through PAA. Hence, the propagation difference between the reflected waves and the character of their interference is altered, and the light-entrapping properties disappear. It should be also mentioned that calcined material is characterized by a different crystallinity, roughness, porosity (i.e., it contains smaller voids in pore walls) and scattering properties, which also contributes to the aforementioned alterations.

The XRD characterization of the thermally cured samples revealed that b-PAA resists crystallization and remains X-ray amorphous up to 850 °C (see Figure S3 in the Supplementary Materials file). This is consistent with the previously reported thermal behavior of transparent PAA formed in sulfuric acid electrolyte, which also shows completely amorphous character at the temperature of 850 °C [29].

**Figure 8:**
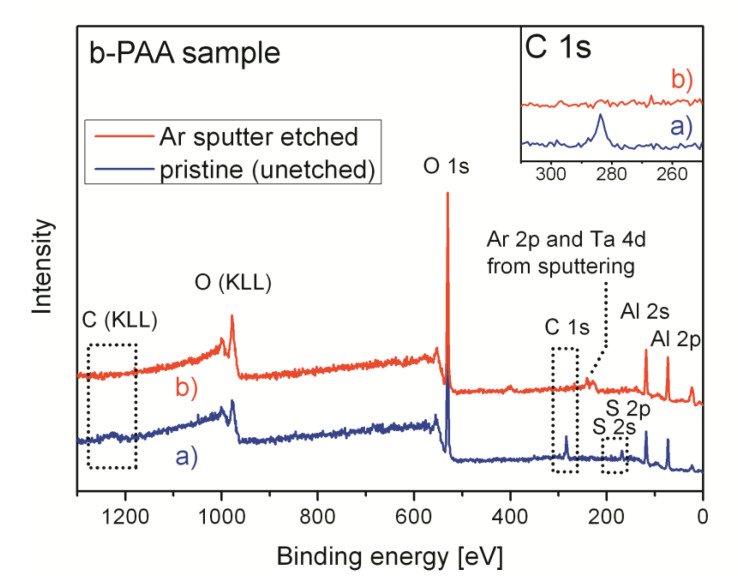
Al Kα (hν=1486.7 eV) survey photoelectron spectra of a b-PAA sample before [blue color, (**a**)] and after [red color, (**b**)] Ar^+^ ion etching. All detected elements are inherent to the typical “sulfuric” anodic alumina composition [see (**a**)]. Casual Ta 4d signals after Ar^+^-ion sputtering [see (**b**)] are due to the XPS-sample preparation procedure (films were pressed to the sample holder by Ta-foil with perforated 2 × 2 mm windows, see the experimental section for explanations). The C 1s photoelectron signal inset demonstrates complete absence of carbon in the bulk of the sample after removal of the thin surface layer (≈30 nm) containing adsorbed species.

The XPS survey spectra of the pristine (uncalcined) b-PAA sample before and after removal of a thin near-surface layer are shown in Figure 8. The surface becomes contamination-free after Ar^+^ sputtering. Only oxygen, aluminum, and sulfur (in smaller amounts than before sputtering) are detected by XPS, whereas no carbon-containing species are observed in the survey spectrum (the C 1s peak completely disappears, which is illustrated by the inset in Figure 8). This suggests that the specimens are free from any casual carbon-containing impurity or graphitic fragments, and only trace amounts of organic substances were adsorbed on the surface.

**Figure 9:**
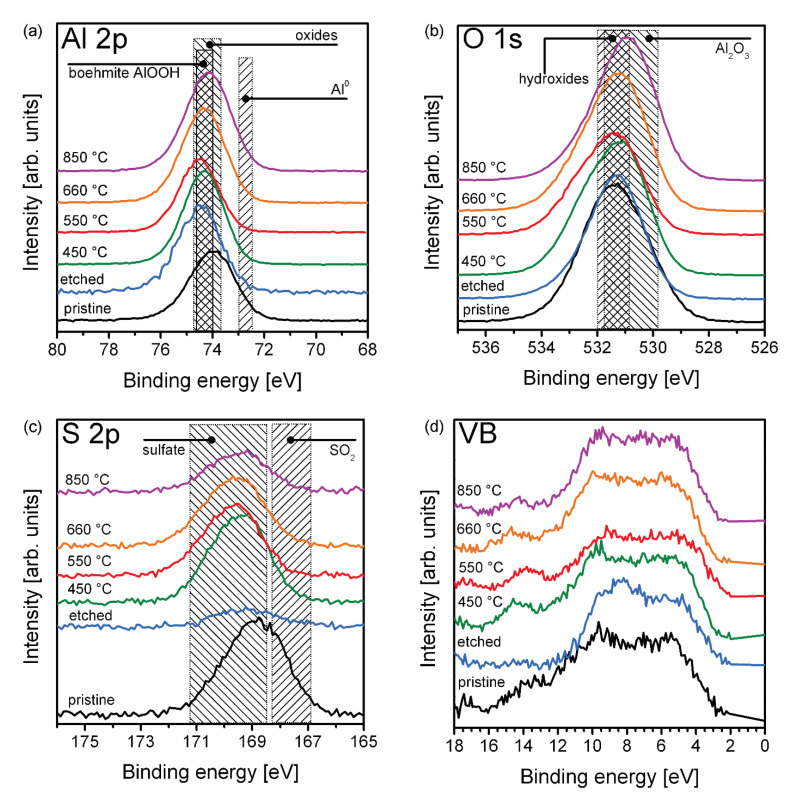
High-resolution Al 2p (**a**)-, O 1s (**b**)-, S 2p (**c**)- and valence band (**d**)- photoelectron spectra of a b-PAA sample calcined at different temperatures. Hatched areas correspond to the binding energy ranges characteristic for (**a**) hydrated boehmite AlOOH, anhydrous oxides, and metallic aluminum, (**b**) aluminum hydroxides and anhydrous stoichiometric oxides, (**c**) sulfate anions and sulfur dioxide.

High resolution Al 2p-, O 1s-, S 2p- and valence band (VB)-photoelectron spectra of the b-PAA samples calcined at different temperatures are shown in Figure 9. The Al 2p photoemissions are situated at the binding energy (*E*_bin_) of around 74.5 eV (Figure 9a), which is inherent for aluminum oxides and hydroxides. Metallic Al^0^ is not detected by XPS (see its binding energy position in Figure 9a). The obtained results agree well with the DTA analysis, which displayed no exothermic signal in the DTA curve that could be interpreted as Al^0^ oxidation, and thus confirmed that the entire aluminum is contained in b-PAA in already oxidized forms. In this regard, the XPS data completely verifies the results indirectly obtained in DTA-analysis. The O 1s photoelectron spectrum (Figure 9b) shows a systematic shift to lower binding energies, i.e., from the values corresponding to hydrated forms of alumina towards water- and hydroxyl-free. This is also supported by the TGA data, which indicated a smooth and extended dehydroxylation process in the whole temperatures range. The S 2p photoelectron spectrum (Figure 9c) gives insight into the distribution of sulfate in the b-PAA layers, as well as into the interplay between sulfate admixture and optical darkening. The preferred surface location of sulfur ions, as compared to the bulk, is supported by our results which demonstrate vanishing of S 2p photoemission after Ar^+^ sputtering. Taking into account the rate of Ar-sputtering, the estimated thickness of S-containing layer is ≈30 nm. The results obtained fully agree with the well-known two-region morphology of a PAA cell where the rich anion contaminated region is situated below the pore surface, whereas the inner framework of PAA consists of relatively pure alumina [35]. The outer anion-containing layer, which also includes the pore interiors close to the pore openings, is removed upon Ar^+^ bombardment. Thus, the inner anion-free part becomes visible for XPS. However, the S 2p signal for Ar^+^-unsputtered samples can be observed for the complete range of the calcination temperatures. This supports the idea that the drastic color changes in b-PAA after 550–660 °C (see Figure 7) are not related to the variations in sulfate content, and can be very likely attributed solely to structural transformations in anodic alumina. The valence band (VB) structure photoemission (Figure 9d) exhibits the spectral features typical for aluminum oxides. There are two pieces of evidence that point at the non-metallic character of the b-PAA layers. The first is the Fermi level position (*E*_F_) at ≈ 2.8 ± 0.6 eV from the valence band maximum (VBM) of all studied samples, and the second is the absence of the spectral features at the *E*_F_.

**Figure 10:**
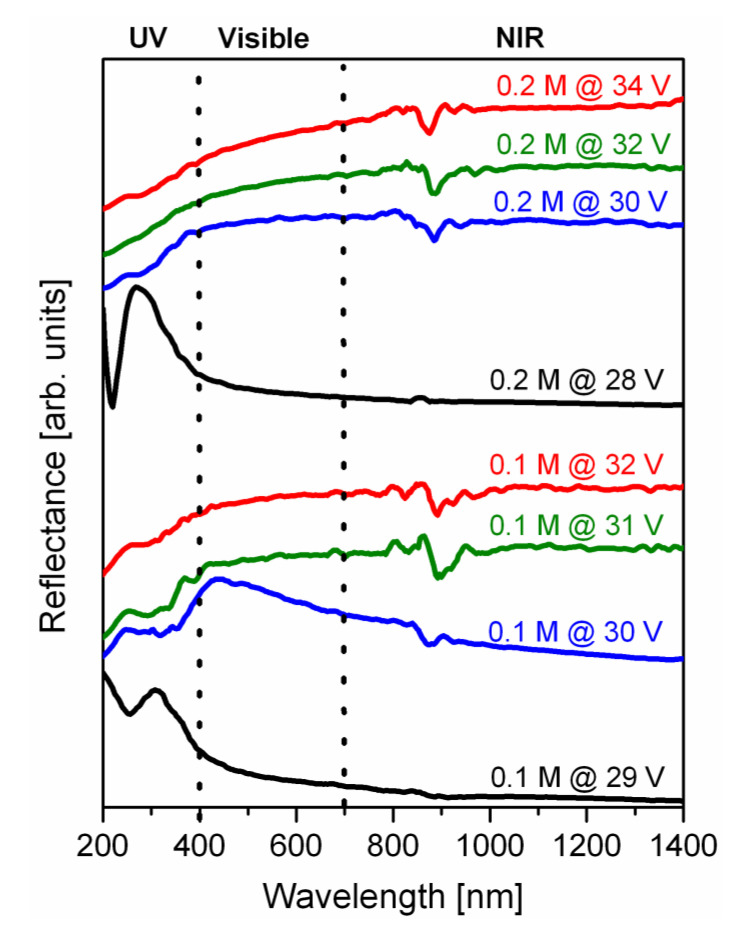
Reflectance spectra of PAA and b-PAA samples obtained in two series of experiments with different 0.1 M and 0.2 M sulfuric acid electrolyte concentrations.

In order to follow the changes in spectral response upon the transition from reflective PAA to light absorbing b-PAA layers, their optical properties were characterized preliminarily by reflectance measurements (UV/Vis/NIR spectrometry, see Figure 10). It must be noted, however, that anodic alumina always has variable morphology and non-stoichiometric composition depending on the employed anodizing conditions, which obviously also changes its optical properties in every individual case. On the one hand, such variation freedom can facilitate further application-specific engineering of b-PAA layers and tuning of their properties. On the other hand, such composition-variability does not permit to acquire any universal analysis data, fundamentally valid for any synthesized alumina type. Therefore, our reflectance spectra can only roughly demonstrate the trends in optical properties changes upon the PAA/b-PAA transition, but they cannot serve as an accurate reference for samples synthesized under different experimental conditions for understandable reasons. Irrespective of the possible involved mechanisms, one apparent common difference can be experimentally seen between PAA and b-PAA samples: whereas transparent PAA layers demonstrate a high spectral reflectance (above 50–55%) within the UV-light region, the reflectance of all optically black b-PAA samples is significantly quenched at wavelengths below *λ* = 400 nm. As the anodizing voltage increases and the qualitative transition from PAA towards b-PAA occurs, a red shift of these bands maxima together with a significant drop of the reflected light intensity can be observed. This is illustrated by the examples of b-PAA synthesized in 0.1 M H_2_SO_4_ at 30 V (the band maximum shifted from ≈310 nm to ≈440 nm) and in 0.2 M H_2_SO_4_ at 30 V (from ≈270 nm to ≈380 nm, which is accompanied by a drastic drop of intensity, so that the signal almost disappears). In all further studied cases where b-PAA layers were synthesized at voltages above 30 V (irrespective of the electrolyte concentration), their low reflectivity was maintained in the UV-light region, and only moderate increase (small slope curves) could be recorded in the range of visible and infrared wavelengths. It should be noted that both the PAA and b-PAA layers show low reflectance in the visible region. However, the explanations of such a measured effect can be different for both cases. While PAA samples are highly transparent for the visible light due to their insulator properties, the photonic light entrapping mechanism can possibly play a leading role in the opaque b-PAA layers. The version of the UV/Vis/NIR spectrometry plots which are extended towards the direction of “thermal infrared” wavelengths and include the measurements in the short-wavelength infrared (SWIR) region are demonstrated in Figure S4 of the Supplementary Materials file. All b-PAA samples demonstrate a flat spectral response within the extended IR-wavelengths region, and thus behave similarly to the standard low-reflectivity (“black”) surface treatments, used in the aerospace technology and for optical applications (manufacturing of telescope housings etc.).

## 4. Conclusions

To summarize, we have developed a firm scientific and technological basis for electrochemical manufacturing of homogeneous optically black pigment-free alumina coatings. The new method offers the potential for application-specific tailoring of their light absorbing properties. The extensive analysis carried out by using different microscopic, spectroscopic, diffraction and thermal analysis methods shows that the blackening in new opaque layers arises due to the structural effects, but not due to the variable composition or accidental admixture. The new observations may help to clarify some of the structural coloration aspects in porous anodic alumina layers in a broader sense, but at the same time they raise new complex questions. For instance, it would be logical to ask why not every known disordered PAA sample shows optical darkening. This is very likely because the photonic light entrapping effect arises not only from the randomness in pore layout, but from a complex combination of different factors. First, the material has to incorporate well-defined and highly uniform light scattering elements (that is to say, straight same-size cylindrical nanochannels throughout our inverse photonic 2D system). Second, the distances between light scattering elements (not necessarily directly neighboring) which contribute to the destructive interference must correlate with half the wavelength from the visible part of the spectrum. Third, the evolution of the microstructure parameters of PAA may also take place under the influence of the film growth conditions, and contribute to the disorder-induced light scattering properties. It is common knowledge that PAA cells consist of compact and polycrystalline (particle size <2.5 nm) regions with different levels of electrolyte contamination [35,36]. In other words, every PAA cell contains additional boundaries between solid phases with different refractive indices, which make the resulting light dispersion picture even more complicated. Previously, we have demonstrated that disordered PAA films formed in 0.1 M and 0.2 M sulfuric acid solutions at smaller voltages exhibit a spongy morphology with largely irregular pore sizes and shapes, rather than a well-defined uniform-size cell structure [15]. In addition, there was a larger degree of orientational disorder in nanochannels due to the constant process of pore splitting and, consequently, shortening of the sections where channels are organized in parallel. Such layers displayed no structure-related coloration effect because their morphological parameters and the type of randomness possibly did not meet the criteria for a desired 2D photonic system. In addition, the roughly estimated inter-pore spacing in disordered PAA synthesized under smaller voltages did not correlate with the visible wavelengths and thus violated the rules under which the photonic effects (if any take place) can be pronounced. From this earlier reported example, we can see that the structural coloration is a cumulative, synergistic effect of multiple factors, which need to be successfully combined within such complicated system as porous alumina (and that is what happens in the b-PAA samples presented in this current study).

Further developing of our new proposed synthesis approach and optimization of “controlled aluminum burning” experiments may give rise to the new emerging class of materials, which can be potentially useful for stray light reduction or passive cooling of different systems. These materials can be a cost-effective alternative to the currently employed ion-beam textured or black etched surfaces. They can also be used as a completely inorganic equivalent of the optically black paints, which would help to improve lightfastness and to overcome the chemical stability problems (so-called “outgassing”, or release of volatile organic compounds from paint). It is worth mentioning that the narrow applied voltage ranges in which spontaneous formation of long-range ordered PAA occurs (so-called “mild” and “hard” anodizing regimes, “MA” and “HA”), as well as the ∆*U* intervals corresponding to the formation of disordered laminas, are by now amply documented for different electrolytes. A considerable amount of accumulated experimental data, as well as thoroughly developed theoretical models describing the evolution patterns in self-ordered PAA depending on ∆*U* [37], offer a lot of scope for design of novel photonic coatings with tunable structural effects.

## Data Availability

The data presented in this study are available on request from the corresponding author.

## Supplementary Materials

The following are available online at https://www.mdpi.com/article/10.3390/ma14195827/s1, Figure S1: comparison of photographs of a single b-PAA lamina and a stack of translucent PAA laminas of comparable thickness, Figure S2: a photograph of a highly transparent well-ordered PAA lamina, Figure S3: X-ray diffraction analysis performed for pristine and calcined b-PAA samples (450–850 °C), Figure S4: UV/Vis/NIR spectrometry analysis results for PAA and b-PAA samples including measurements in short-wavelength infrared (SWIR) region.
