# Supplementary material for: A Strategy towards Light-Absorbing Coatings Based on Optically Black Nanoporous Alumina with Tailored Disorder
Source: Materials (Basel). 2021 Oct 5;14(19):5827. doi: 10.3390/ma14195827 (PMC8510353; doi:10.3390/ma14195827)
Supplement: Supplementary file 1 [file materials-14-05827-s001.zip › materials-1399276-supplementary.pdf]

A Strategy towards Light-Absorbing Coatings Based on Optically Black Nanoporous Alumina with Tailored Disorder

Mikhail Pashchanka [1,*] and Gennady Cherkashinin [2]

[1] Department of Chemistry, Eduard-Zintl-Institute, Technical University of Darmstadt, Alarich-Weiss-Straße 12, 64287 Darmstadt, Germany
[2] Institute of Materials Science, Technical University of Darmstadt, Alarich-Weiss Straße 2, 64287 Darmstadt, Germany; gennady.cherkashinin@tu-darmstadt.de
* Correspondence: mikhail.pashchanka@gmail.com

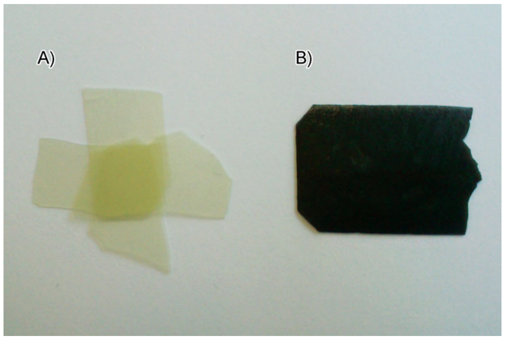

Figure S1. Comparison of the visual properties of (**A**) – stacked translucent porous alumina (a single layer thickness ≈ 70 μm) and (**B**) – black opaque alumina of the equivalent layer thickness (280-300 μm).

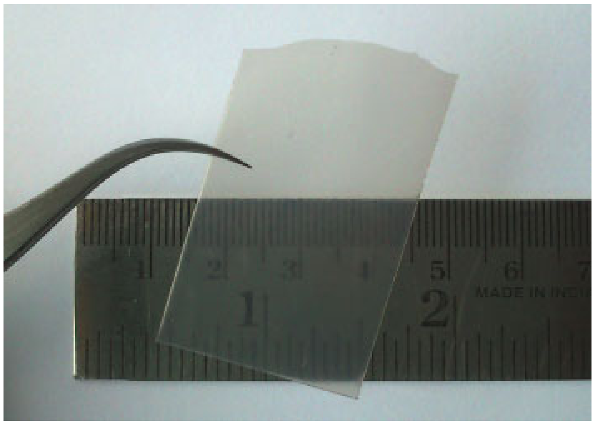

Figure S2: A photograph of the highly ordered and transparent PAA obtained from 0.3 M H_2SO_4 at ΔU = 27 V.

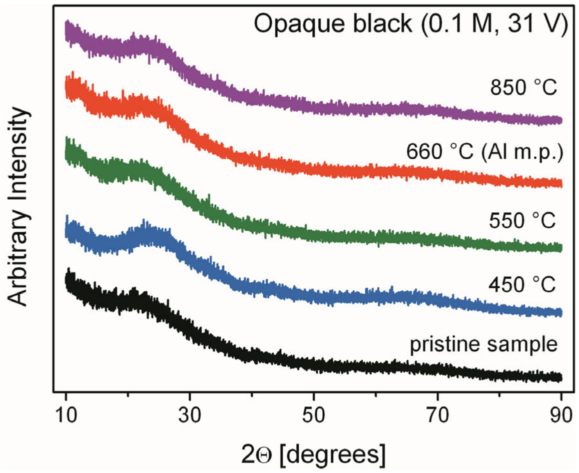

Figure S3: The XRD characterization of the thermally cured black opaque anodic alumina samples.

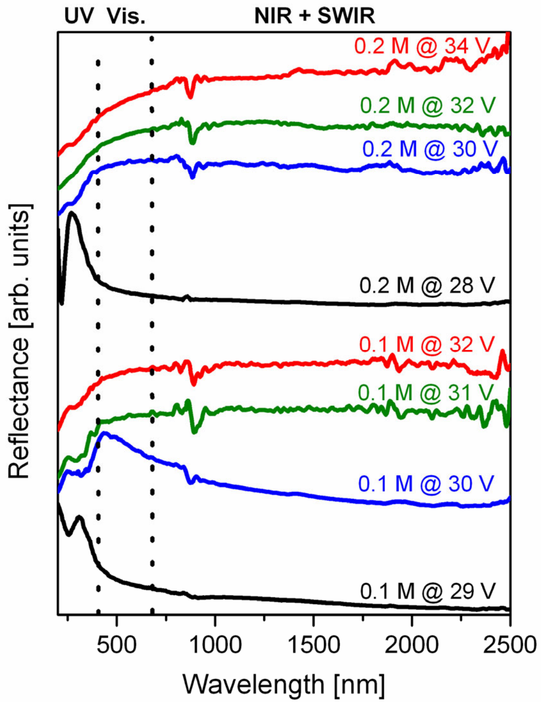

Figure S4: UV/Vis/NIR spectrometry results for PAA and b-PAA samples, which cover the UV, visible, near infrared (NIR) and short-wavelength infrared (SWIR) regions.
